# Apatinib, a novel VEGFR-2 tyrosine kinase inhibitor, for relapsed and refractory nasopharyngeal carcinoma: data from an open-label, single-arm, exploratory study

**DOI:** 10.1007/s10637-020-00925-2

**Published:** 2020-05-03

**Authors:** Ling Li, Fei Kong, Lei Zhang, Xin Li, Xiaorui Fu, Xinhua Wang, Jingjing Wu, Fangwen Zhang, Liangliang Ren, Mingzhi Zhang

**Affiliations:** 1 Department of Oncology, The First Affiliated Hospital of Zhengzhou University, Zhengzhou, 450052 Henan China; 2 Lymphoma Diagnosis and Treatment Center of Henan Province, Zhengzhou, 450052 Henan China

**Keywords:** Apatinib, Efficacy, Nasopharyngeal carcinoma, Targeted therapy

## Abstract

*Purpose* Apatinib, a new tyrosine kinase inhibitor targeting vascular endothelial growth factor receptor-2, has shown promising efficacy against several solid cancers, but evidence of its efficacy against relapsed and refractory nasopharyngeal carcinoma is limited. We investigated the efficacy and safety of apatinib for relapsed and refractory nasopharyngeal carcinoma in an open-label, single-arm, phase II clinical trial. Fifty-one patients with relapsed and refractory nasopharyngeal carcinoma in the First Affiliated Hospital, Zhengzhou University, who met the inclusion criteria were enrolled in the study. All patients received apatinib at an initial dose of 500 mg daily (1 cycle = 28 days). The primary and secondary endpoints were overall response rate, progression-free survival, and overall survival. We evaluated treatment effects and recorded apatinib-related adverse events by performing regular follow-ups and workup. The overall response rate (complete and partial responses) was 31.37% (16/51). The median overall survival and progression-free survival were 16 (95% CI, 9.32–22.68) and 9 months (95% CI, 5.24–12.76), respectively. Most patients tolerated treatment-related adverse events of grades 1 and 2; hypertension (29, 56.86%), proteinuria (25, 49.02%), and hand–foot syndrome (27, 52.94%) were the most common adverse events. There were no treatment-related deaths. Apatinib showed good efficacy and safety in patients with relapsed and refractory NPC.

## Introduction

Nasopharyngeal carcinoma (NPC) is a common malignant tumor of the head and neck with a distinct regional and racial prevalence in southern China, especially among people of Cantonese ancestry. Radiotherapy is the main treatment for NPC [1, 2]. Despite intensive treatment, 30%–40% of patients with NPC show progressive disease [3, 4], and the treatment of patients with relapsed and refractory NPC remains a major challenge. Although palliative chemotherapy can confer median progression-free survival (PFS) for 3–9 months in patients with a recurrent disease, the overall survival (OS) remains low [5]. Hence, patients with relapsed and refractory NPC are recommended to participate in clinical trials.

Angiogenesis is an important step in the development of several malignant tumors. Apatinib, a new vascular endothelial growth factor receptor-2 (VEGFR-2) tyrosine kinase inhibitor, selectively targets intracellular ATP-binding site and has shown efficacy against various solid tumors, especially advanced gastric carcinoma [5–8]. In a previous study [9], VEGF was overexpressed in more than 60% of clinical biopsy samples of NPC. Co-expression of tumor VEGF and hypoxia-related growth factors in NPC is associated with poor prognosis, and it serves as potential evidence to explore the effectiveness of apatinib against relapsed and recurrent NPC. However, clinical data to help assess the antitumor activity against recurrent and refractory NPC are limited. Hence, in the present study, we evaluated the safety and efficacy of apatinib in 51 patients with relapsed and refractory NPC.

## Materials and methods

### Ethical statement

The patient data used in the study were anonymized. All procedures were in agreement with the Declaration of Helsinki.

### Patients and eligibility criteria

Fifty-one patients in the First Affiliated Hospital of Zhengzhou University, Henan, China, were enrolled between December 2016 and January 2019. The inclusion criteria were as follows: 1) patients aged between 18 and 70 years with an Eastern Cooperative Oncology Group (ECOG) performance status of 0–3; 2) patients with histologically confirmed NPC who did not respond to the first-line platinum-containing chemotherapy and second-line single or combined chemotherapy before apatinib treatment according to the National Comprehensive Cancer Network guideline; and 3) patients with at least one measurable lesion according to the Response Evaluation Criteria in Solid Tumors (RECIST) and with acceptable hematologic, hepatic, and renal functions. Patients were excluded if they had other malignant tumors; cardiac insufficiency or arrhythmia; uncontrolled complications such as diabetes mellitus, coagulation disorders, and urine protein ≥ ++; or were pregnant or breastfeeding.

### Study design

Each patient received apatinib 500 mg at 30 min after lunch, at the same time, once daily as an initial dose until disease progression or intolerable toxicity was noted (1 treatment cycle = 28 days). Dose interruptions and reductions were allowed in cases of grade 3/4 toxicities. For each treatment cycle, interruptions were allowed for a maximum of 7 days continuously or cumulatively, and dose reductions from 500 to 375 mg daily and then to 250 mg daily were allowed for a maximum of two times. For intolerable grade 2 toxicities, one dose reduction was considered when the investigators deemed necessary.

### Assessments and statistical analysis

Overall response rate (ORR) was defined as the ratio of patients achieving a complete response (CR) or partial response (PR) according to RECIST1.1 [10, 11]. PFS was defined as the time from enrollment to documentation of disease progression and OS was defined as the time from assignment to death from any cause. Both PFS and OS were estimated using the Kaplan–Meier method with 95% confidence intervals (CIs). Baseline evaluation before treatment included a physical examination, the ECOG status assessment, antecedent blood pressure measurement, complete blood count (CBC), blood chemistry panel, coagulation function test, routine urinalysis, routine stool test consisting of occult blood (OB) testing, echocardiography (ECG), and baseline lesion evaluation by contrast-enhanced computerized tomography (CT). The following tests were performed at follow-up: physical examination, CBC, biochemical profiling, and dynamic contrast-enhanced CT or magnetic resonance imaging (MRI). Treatment efficacy was evaluated after every two cycles by contrast-enhanced CT. Adverse events (AEs) were assessed according to the Common Terminology Criteria for Adverse Events (CTCAE) version 5.0 and related data were collected during outpatient clinic visit and follow-up [12].

## Results

### Baseline characteristics

Fifty-five patients with relapsed and refractory NPC were registered in the study after obtaining signed informed consent (Table 1). During the screening stage, one patient did not meet the inclusion criteria and three patients were excluded from the per-protocol population because their post-baseline efficacy assessment data were not available. The remaining 51 patients were included in the safety and activity analyses (Table 1).

**Table 1:** Baseline characteristics of 51 patients

Characteristics	Number of patients (%)
Age, years	
Median age	50
Range	(18 to 70)
Sex	
Male	43 (84.31)
Female	8 (15.69)
ECOG performance status	
≤2	31 (60.78)
>2	20 (39.22)
Pathological type (WHO)	
WHO I	9 (17.65)
WHO II/III	42 (82.35)
EBER	
Negative	10 (19.61)
Positive	41 (80.39)
bFGF	
Positive	12 (23.53)
Negative	39 (76.47)
Ki67	
<25%	10 (19.61)
>25%	41 (80.39)
Hb (g/L)	
≥120	39 (76.47)
<120	12 (23.53)
Lactate dehydrogenase (IU/L)	
≤245	35 (68.63)
>245	16 (31.37)
Alkaline phosphatase (IU/L)	
≤110	30 (58.82)
>110	21 (41.18)
T stage (UICC/AJCC 8th edition)	
T1–2	7 (13.73)
T3–4	44 (86.27)
N stage (UICC/AJCC 8th edition)	
N1–2	10 (19.61)
N3–4	41 (80.39)
Clinical stages	
III	20 (39.22)
IV	31 (60.78)
Distant metastasis	
Yes	39 (76.47)
No	12 (23.53)
Radiotherapy of the primary tumor	
Yes	48 (94.12)
No	3 (5.88)
Metastatic lesion	
Bone	20 (39.22)
Liver	11 (21.57)
Lung	13 (25.49)
Distant lymph nodes, mediastinum, and others	7 (13.73)

Abbreviations: ECOG, Eastern Cooperative Oncology Group; LDH, lactate dehydrogenase; Hb, hemoglobin; ALP, alkaline phosphatase

### Efficacy

**Fig. 1:**
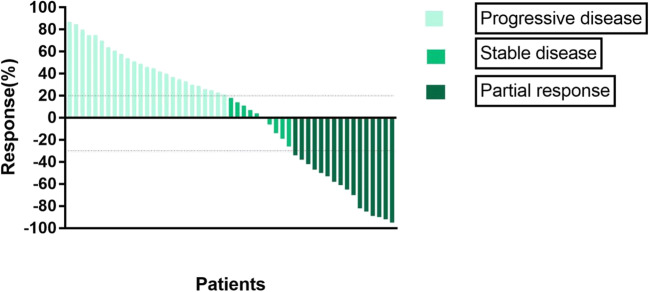
Waterfall plot for the best percentage change in target lesion size

**Fig. 2:**
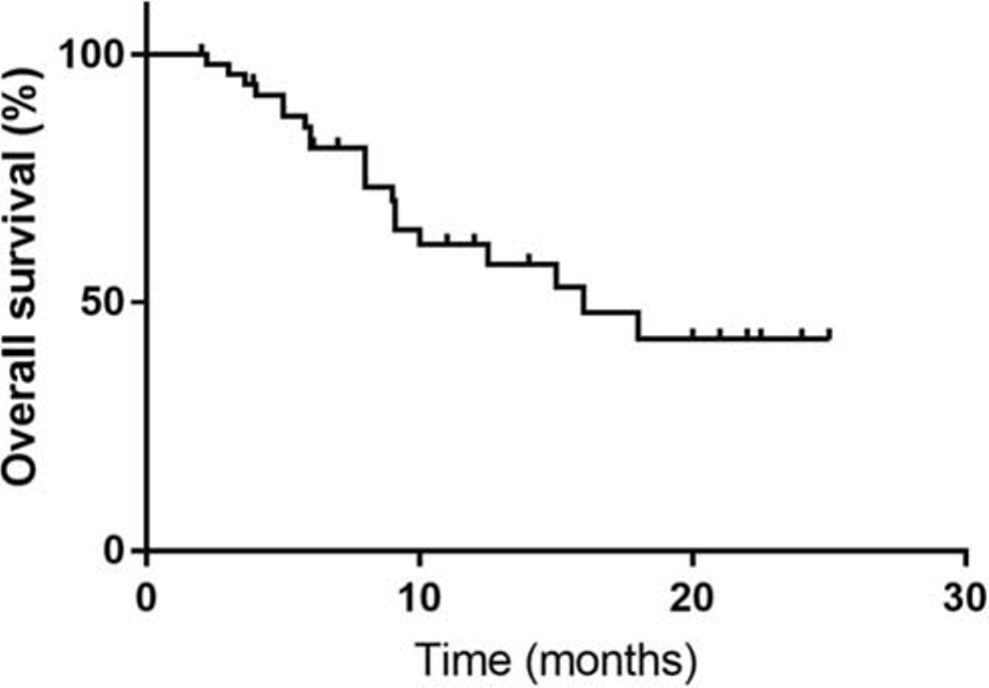
Kaplan–Meier graph for overall survival (*n* = 51)

**Fig. 3:**
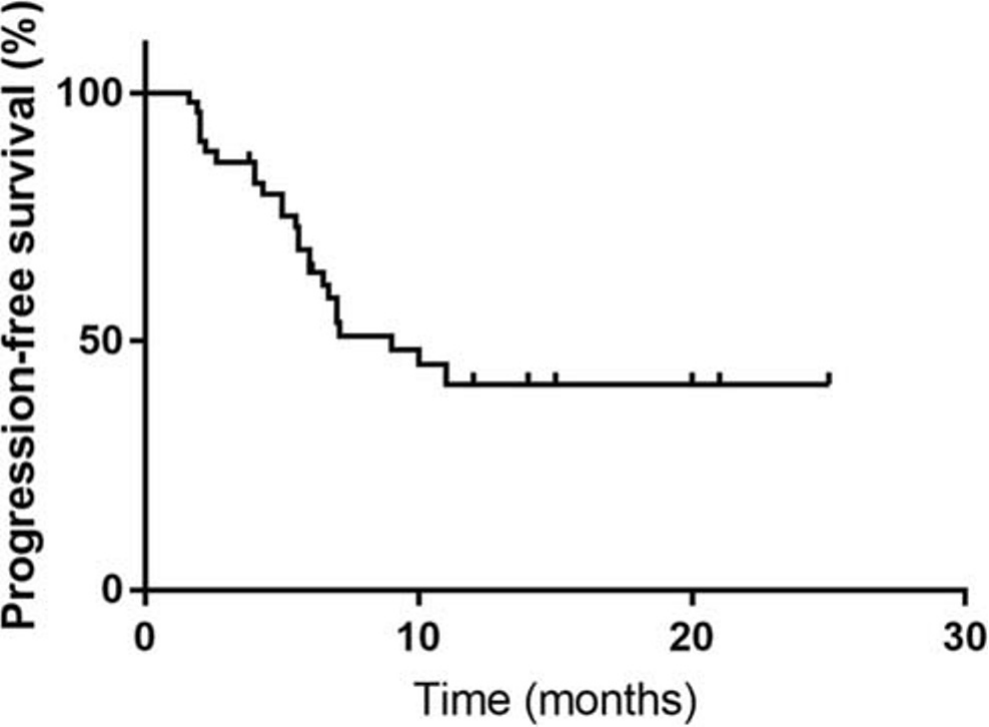
Kaplan–Meier graph for progression-free survival (n = 51)

The data collection cut-off time for the primary analysis was May 2019. By the last follow-up, 16 (31.37%) patients had achieved ORR, with 0 cases of CR and 16 cases (31.37%) of PR (Table 2). Twenty-five of the 51 (49.02%) patients showed disease progression, and five of them showed progression during the initial two-cycle evaluation. The median OS was 16 months (95% CI, 9.32–22.68) and the median PFS was 9 months (95% CI, 5.24–12.76) (Figs. 1, 2 and 3). Twenty patients (39.22%) died before the cutoff date; among these, eight (15.69%) showed PR and 12 (23.53%) showed disease progression. Twenty of the remaining patients showed tumor shrinkage from the baseline findings at their last follow-up. The percentage changes from the baseline are shown in Fig. 1.

**Table 2:** Treatment responses

Effective evaluation	N (%)
CR	0
PR	16 (31.37)
SD	10 (19.61)
PD	25 (49.02)
ORR (CR + PR)	16 (31.37)
DCR (CR + PR + SD)	26 (50.98)

CR, complete response; PR, partial response; SD, stable disease; PD, progressive disease; ORR, overall response rate; DCR, disease control rate

### Safety

The incidence of AEs of any grade, regardless of causality, was 100%. These included hematological and non-hematological toxicities (Table 3). Notably, simultaneous occurrence of multiple AEs in patients was common. Most patients showed treatment-related AEs of grades 1–2, with proteinuria (25 patients, 49.02%), hypertension (29 patients, 56.86%), and hand–foot syndrome (27 patients, 52.94%) being the most common. AEs of grades 3–4 were noted in 18 patients. Fatigue was a common symptom among patients with leucopenia. One patient was admitted to the hospital to receive treatment for severe hemorrhinia, which was considered possibly treatment related. There was no grade 5 AE.

**Table 3:** Major treatment-related adverse events, n (%)

Adverse events	Grade 1–2, n (%)	Grade 3, n (%)	Grade 4, n (%)	Grade 5, n (%)	ALL, n (%)
**Non-hematologic**					
Hypertension	23 (45.10)	3 (5.88)	3 (5.88)	0	29 (56.86)
Hand–foot syndrome	25 (49.02)	2 (3.92)	0	0	27 (52.94)
Proteinuria	24 (47.06)	1 (1.96)	0	0	25 (49.02)
Mucositis	10 (19.61)	0	0	0	10 (19.61)
Fatigue	18 (35.29)	1 (1.96)	1 (1.96)	0	20 (39.22)
Myalgia/arthralgia	5 (9.80)	0	0	0	5 (9.80)
Hyperbilirubinemia	1 (1.96)	0	0	0	1 (1.96)
Aminotransferase increased	2 (3.92)	0	0	0	2 (3.92)
Vomiting	9 (17.65)	1 (1.96)	0	0	10 (19.61)
Erythra	2 (3.92)	0	0	0	2 (3.92)
Nausea	7 (13.73)	1 (1.96)	0	0	8 (15.69)
Headache	6 (11.76)	2 (3.92)	0	0	8 (15.69)
Pain	3 (5.88)	0	0	0	3 (5.88)
Alopecia	2 (3.92)	0	0	0	2 (3.92)
hemorrhage	7 (13.73)	0	2 (3.92)	0	9 (17.65)
**Hematologic**					
Leucopenia	7 (13.73)	0	0	0	7 (13.73)
Neutropenia	18 (35.29)	1 (1.96)	0	0	19 (37.25)
Thrombocytopenia	6 (11.76)	0	0	0	6 (11.76)
Anemia	15 (29.41)	1 (1.96)	1 (1.96)	0	17 (33.33)

### Dose adjustments

Dose adjustments were necessary (refer to Study design) for 31 (60.78%) patients. For 23 patients (45.10%), one dose reduction from 500 mg per day to 375 mg was required, and for eight patients (15.69%), the dose was reduced to 250 mg per day. Treatment was temporarily interrupted for 27 patients (52.94%) and was resumed after an interval of fewer than 7 days or rational dose adjustments were performed (Table 4). Treatment was interrupted permanently owing to grade 4 hypertension in 4 patients (7.84%) and due to severe hemorrhinia in 1 patient. The symptoms in these patients were resolved after support treatment.

**Table 4:** Dose adjustments (n, %)

**Dose interrupted permanently**	4 (7.84)
**Dose interrupted temporarily**	27 (52.94)
**Dose Adjustments**	
No dose adjustments (500 mg)	20 (39.22)
375 mg per day	23 (45.10)
250 mg per day	8 (15.69)

## Discussion

Cisplatin combined with 5-fluorouracil is a common first-line regimen for advanced NPC, with the associated response rates of 66%–78% and median survival of 11–14 months [13–15]. Taxane- or gemcitabine-based doublet combination regimen has also been commonly employed, with a response rate of 22%–75% [16–18]. Treatment for patients with recurrent NPC is challenging because there is no standard second-line treatment regimens after the failure of the first-line platinum-based chemotherapy. This group of patients represents those with the most urgent unmet therapeutic need.

Molecular-targeted therapy has been broadly explored and evaluated against one or several genetic mutations, aberrant growth factor pathways, or angiogenesis in NPC. To the best of our knowledge, this is the first study to report the efficacy and safety of apatinib for treating relapsed and refractory NPC. As an oral anti-angiogenesis drug for advanced tumors, apatinib showed potential in our study. We observed objective responses in 31.37% patients, achieving a median PFS of 9 months and a median OS of 16 months, superior to those associated with several popular novel agents (Table 5). Nivolumab is a human monoclonal antibody that targets programmed death receptor-1 (PD-1); it has been approved for the treatment of various types of cancer in several countries. Early-stage clinical trials of anti-PD-1 therapies have shown promising outcomes with objective response rates (ORRs) of 21%–34% in recurrent or metastatic NPC [19, 20]. Axitinib, another VEGFR inhibitor, has also been reported to help achieve an ORR of 19% among 37 evaluable patients, including 1 who showed PR, 6 who showed unconfirmed PR, and 22 who showed a stable disease. However, the median time to progression was 5.0 months and median OS was 10.4 months, indicating an inferior curative effect compared with that of apatinib [21]. Adoptive T cell therapy has emerged as a strategy to treat human cancers. Despite a preferable median OS of 38.1 months [22], which superior to that observed in our study, the feasibility and generalization of adoptive T cell therapy are limited by extremely high cost and complexity of the associated laboratory techniques. Thus, significant challenges in solid cancer management remain.

**Table 5:** Comparison of clinical characteristics of apatinib and other novel antigens for relapsed and refractory NPC

Drug	Study	N	ORR	PFS	OS
Axitinib	Hui et al.	37	19%	5 months	10.4 months
Nivolumab	Ma et al. [20]	32	13%	3.5 months 3-month PFS, 64.2%	3-month OS, 87.5% OS was not reached
Pazopanib	Lim et al. [29]	33	6.1%	4.4 months	1-year OS, 32%
Interleukin-2	Chi et al. [30]	14	0	–	9 months
Adoptive T cell therapy	Smith et al. [22]	29	–	5.5 months	38.1 months

Angiogenesis is mainly affected by the microenvironment, and VEGFR is one of the most important angiogenesis factors. Apatinib acts by selectively competing for ATP-binding sites of VEGFR-2. VEGFR-2 is autophosphorylated at its carboxyl terminal tail and kinase insert region, leading to the activation of its kinase activity and binding of phospholipase C-γ plus the adaptor molecules TSAd, Sck, and Shb [23, 24]. This triggers the downstream RAS-RAF-MEK-ERK and PI3K pathways [25]. The vital role of angiogenesis in malignancies has been confirmed. However, to the best of our knowledge, there are no studies on the efficacy and safety of VEGF-2 inhibitors for relapsed and refractory NPC.

Hypertension, proteinuria, and hand–foot syndrome are regarded as the most common AEs of anti-angiogenic agents (Table 3). In our study, most cases of hypertension, proteinuria, and hand–foot syndrome were of grades 1 and 2, with incidences of 56.86%, 49.02%, and 52.94%, respectively. These findings were generally consistent with the results of previous studies on other solid cancers [26, 27]. It is worth noting that one patient showed severe hemorrhinia, possibly related to the treatment, which could indicate that VEGFR inhibitor drugs may increase bleeding risk in patients. This could be due to drug-induced platelet dysfunction and reduced synthesis of vascular endothelial tissue factor, damaging vascular integrity. VEGF can stimulate the proliferation of vascular endothelial cells, maintain the integrity of blood vessels, and ensure the normal regulation of coagulation function. Inhibition of the VEGFR transduction pathway can reduce the regeneration ability of vascular endothelial cells, expose procoagulant phospholipids under the matrix, and cause platelet dysfunction, leading to hemorrhage or thrombosis [28].

Apatinib administered orally, without the need for hospitalization of patients or an infusion pump for administration, might result in improved patient compliance and economic feasibility. To the best of our knowledge, this is the first study to evaluate the efficacy and safety of apatinib in patients with relapsed and refractory NPC. The antitumor effects and reverse drug resistance may be improved by combining apatinib treatment with chemotherapy or other targeted drugs, including anti-angiogenesis agents with different action mechanisms. However, this study had some limitations. This was a single-arm, retrospective study with no control group for comparison, and thus selection bias could not be ruled out because of the non-randomized design. Second, the study population was relatively small. Nonetheless, based on our results, apatinib appears to be safe and highly efficacious against relapsed and refractory NPC, which strongly indicates the need for further research to confirm its efficacy against nasopharyngeal cancer.
